# New and little-known species of *Tipula* Linnaeus (Diptera, Tipulidae) from Laos, with a new synonym

**DOI:** 10.3897/zookeys.982.49781

**Published:** 2020-11-02

**Authors:** Bing Zhang, Jinlong Ren, Yan Li, Ding Yang

**Affiliations:** 1 Department of Entomology, College of Plant Protection, China Agricultural University, Beijing 100193, China China Agricultural University Beijing China; 2 College of Plant Protection, Shenyang Agricultural University, Shenyang, 110866, China Shenyang Agricultural University Shenyang China

**Keywords:** biodiversity, description, distribution, systematics, taxonomy

## Abstract

Only seven species of the genus *Tipula* Linnaeus were previously known to occur in Laos. Here one new species is added to the fauna of Laos, Tipula (Nippotipula) champasakensis**sp. nov.***Tipula* (unplaced) *cladomera* Alexander, 1936 is designated as a synonym of *T.* (unplaced) *reposita* Walker, 1848, **syn. nov.** A key to species of the genus *Tipula* from Laos is presented.

## Introduction

The genus *Tipula* Linnaeus is a large genus in the family Tipulidae. It is distributed worldwide with 2445 known species and subspecies, of which 1024 taxa are from the Palaearctic Region, 496 taxa from the Nearctic Region, 464 taxa from the Neotropical Region, 112 taxa from the Afrotropical Region, 356 taxa from the Oriental Region, and 30 taxa from the Australasian/Oceanian Region (Oosterbroek 2020). This genus is characterized by the following features: antenna usually 13-segmented, rarely 14-segmented, each segment dilated at base with 4–6 setae except scape, pedicel and first flagellomere; formula of tibial spurs 1–1–2, 1–2–2, 2–1–2 or 2–2–2; wings with two anal veins, A_2_ usually away from inner margin, and cell a_2_ broad; Rs long and originating well before end of Sc_2_; M separated into three veins, cell m_1_ petiolate; m-cu located beyond fork of M (Savchenko 1961; Joseph 1974; McAlpine 1981; Savchenko 1983).

The subgenus Nippotipula Matsumura is a small subgenus in the genus Tipula. It contains 17 known species and subspecies, of which five taxa are from the Palaearctic Region, two taxa from the Nearctic Region, and 15 taxa from the Oriental Region (Oosterbroek 2020). This subgenus is characterized by the following features: formula of tibial spurs 1–2–2; R_4+5_ in a straight line with the Rs (base of R_4+5_ not curving); Rs long, at least twice as long as m-cu (Edwards 1931); abdomen exceeding wings; lobe of gonostylus larger than clasper of gonostylus, and with strong, dense setae.

So far, only the following seven species of the genus *Tipula* were known to occur in Laos (Oosterbroek 2020): T. (Formotipula) laosica Edwards, 1926, T. (F.) melanomera gracilispina Savchenko, 1960 (Zhang et al. 2019), T. (F.) melanomera melanomera Walker, 1848, T. (F.) melanopyga Edwards, 1926, T. (Platytipula) sessilis Edwards, 1921, T. (Schummelia) vitalisi Edwards, 1926, and *T.* (unplaced) *reposita* Walker, 1848. To enrich the number and distribution of craneflies in Laos, we conducted a one-month scientific research of craneflies in Laos in June 2017. Presently, three species of the genus *Tipula*, two known species and one new species, were add to the fauna of Laos. We also designated *T.* (unplaced) *cladomera* Alexander, 1936 as a synonym of *T.* (unplaced) *reposita* Walker, 1848.

So far, only the following seven species of the genus *Tipula* were known to occur in Laos (Oosterbroek 2020): T. (Formotipula) laosica Edwards, 1926, T. (F.) melanomera gracilispina Savchenko, 1960 (Zhang et al. 2019), T. (F.) melanomera melanomera Walker, 1848, T. (F.) melanopyga Edwards, 1926, T. (Platytipula) sessilis Edwards, 1921, T. (Schummelia) vitalisi Edwards, 1926, and *T.* (unplaced) *reposita* Walker, 1848. To enrich the number and distribution of craneflies in Laos, we conducted a one-month scientific research of craneflies in Laos in June 2017. Three species of known species of the genus *Tipula* were found again in Houaphanh, Attapeu, and Champasak provinces. This has made an important contribution to enriching the catalogue of the crane flies of the world. Presently, we add one new species to the fauna of Laos and designate *T.* (unplaced) *cladomera* Alexander, 1936 as a synonym of *T.* (unplaced) *reposita* Walker, 1848.

## Material and methods

The specimens were studied and illustrated with a ZEISS Stemi 2000-c stereomicroscope. Details of coloration were checked in specimens immersed in 75% ethyl alcohol (C_2_H_5_OH). Genitalic preparations of males were made by macerating the apical portion of the abdomen in cold 10% NaOH for 12–15 hours. After examination, it was transferred to fresh glycerine (C_3_H_8_O_3_) and stored in a microvial pinned below the specimen. The specimens studied, which were collected in Laos during June 2017, are deposited in the Entomological Museum of China Agricultural University (**CAU**), Beijing, China.

Some type and non-type material used in this paper were borrowed from the National Museum of Natural History, Smithsonian Institution, Washington, DC, USA (**USNM**) and the Natural History Museum, London, UK (**BMNH**). Unfortunately, specimens of two species previously recorded from Laos were unavailable for study, *T. vitalisi* and *T. laosica*. Therefore, any comparisons/characters mentioned in the key and elsewhere where based on the previously published descriptions of these species.

The morphological terminology mainly follows McAlpine (1981), Alexander and Byers (1981), and Tangelder (1983). Terminology of the male hypopygium follows Ribeiro (2006) and Frommer (1963).

## Taxonomy

### Key to species (dry material) of genus *Tipula* from Laos

**Table:** 

1	Body velvet black or velvet orange (Figs 1, 11, 15)	**2**
–	Body brownish yellow or brown (Figs 19, 34, 38, 49)	**5**
2	Thorax velvet black (Edwards 1926)	**T. (Formotipula) laosica Edwards, 1926**
–	Thorax velvet orange (Figs 1, 11, 15)	**3**
3	Clasper of gonostylus with two short acute blackened spines (Li et al. 2013: 205, fig. 18)	**T. (F.) melanopyga Edwards, 1926**
–	Clasper of gonostylus complex, beak with a lump near tip, back of beak bearing a sickle-shaped prominence directed anteriorly; dististyle outer surface with an acute blackened spine curved up (Fig. 7)	**4**
4	Clasper of gonostylus broad and short, beak slender (Savchenko 1960: 888, fig. 2)	**T. (F.) melanomera gracilispina Savchenko, 1960**
–	Clasper of gonostylus slender, beak short (Savchenko 1960: 888, fig. 1)	**T. (F.) melanomera melanomera Walker, 1848**
5	Body large, greater than 20 mm; eighth sternite extended backward, posterior margin with deep median notch, two small triangular processes present at bottom of notch (Fig. 25)	**T. (Nippotipula) champasakensis sp. nov.**
–	Body not exceeding 20 mm; eighth sternite not as above	**6**
6	Posterior margin of cell cua_1_ as wide as base (Figs 40, 50)	***T.* (unplaced) *reposita* Walker, 1848**
–	Posterior margin of cell cua_1_ narrower than base (Fig. 37)	**7**
7	Rs a little longer than R_2+3_, relatively straight (Fig. 37)	**T. (Platytipula) sessilis Edwards, 1921**
–	Rs very short, equaling R_2_, which is distinct and oblique (Edwards 1926)	**T. (Schummelia) vitalisi Edwards, 1926**


#### 1. Tipula (Formotipula) melanomeragracilispina

Taxon classificationAnimaliaDipteraTipulidae

Savchenko, 1960

9127DABE-E236-59E2-917F-83F660AE653C

Figs 1–4 , 5–7 , 8–10

Tipula melanomera gracilispina Savchenko 1960: 888. Type locality: China: Yunnan.
Tipula (Formotipula) melanomera gracilispina : Li et al. 2013: 207.

##### Diagnosis.

Hypopygium is blackish with black setae. Posterior margin of ninth tergite has a low U-shaped notch. Posterior margin of eighth sternite has a pair of digitiform appendages. Clasper of gonostylus is complex, broad, and short; beak slender.

##### Redescription.

Male (*n* = 5): Body length 13–15 mm, wing length 14–16 mm, antenna length 3.5–4.0 mm.

**Figures 1–4.**
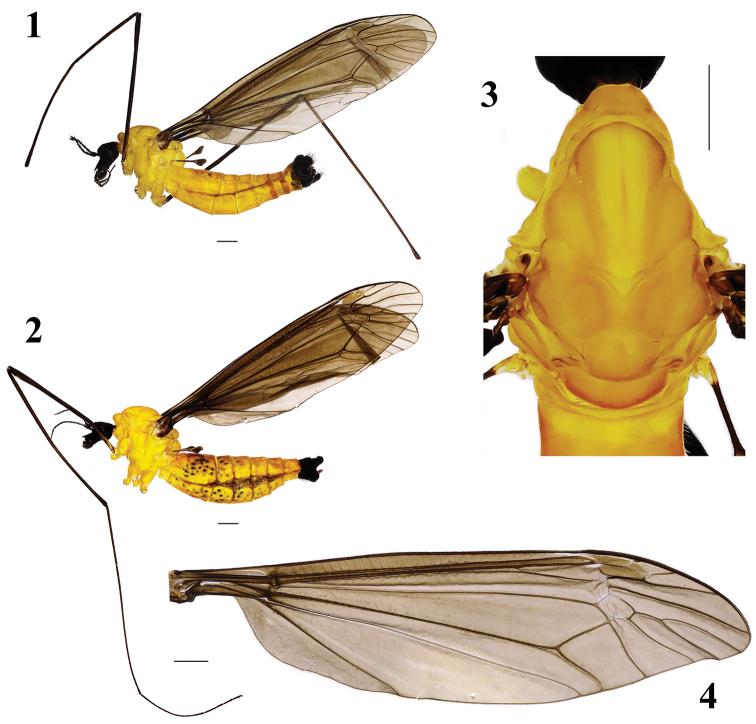
Tipula (Formotipula) melanomera gracilispina Savchenko **1** male habitus, lateral view **2** female habitus, lateral view **3** male thorax, dorsal view **4** male right wing. Scale bar: 1.0 mm.

Head (Figs 1, 3). Mostly velvet black. Eyes dark black. Dorsal part of rostrum brownish black. Setae on head black. Antenna dark brown except scape and pedicel brownish with black setae; palpus brownish grey with black setae.

Thorax (Figs 1, 3, 4). Mainly bright orange. Prescutum orange with yellowish-white pollen; pleuron mostly yellowish orange with yellow setae. Legs: coxae and trochanters grayish brown; femora brownish black; tibiae and tarsi dark brown. Setae on legs black except coxae and trochanters with yellow setae. Wing brownish; pterostigma dark brown with some macrotrichiae; venation brownish black, Rs relatively long, cell m_1_ petiolate (Fig. 4). Halter length approximately 2 mm, halter stem brownish with brown setae; halter brown with black setae.

Abdomen (Fig. 1). Mainly dull orange. Abdominal segments 1–7 orange with brownish setae. Hypopygium blackish with black setae.

**Figures 5–7.**
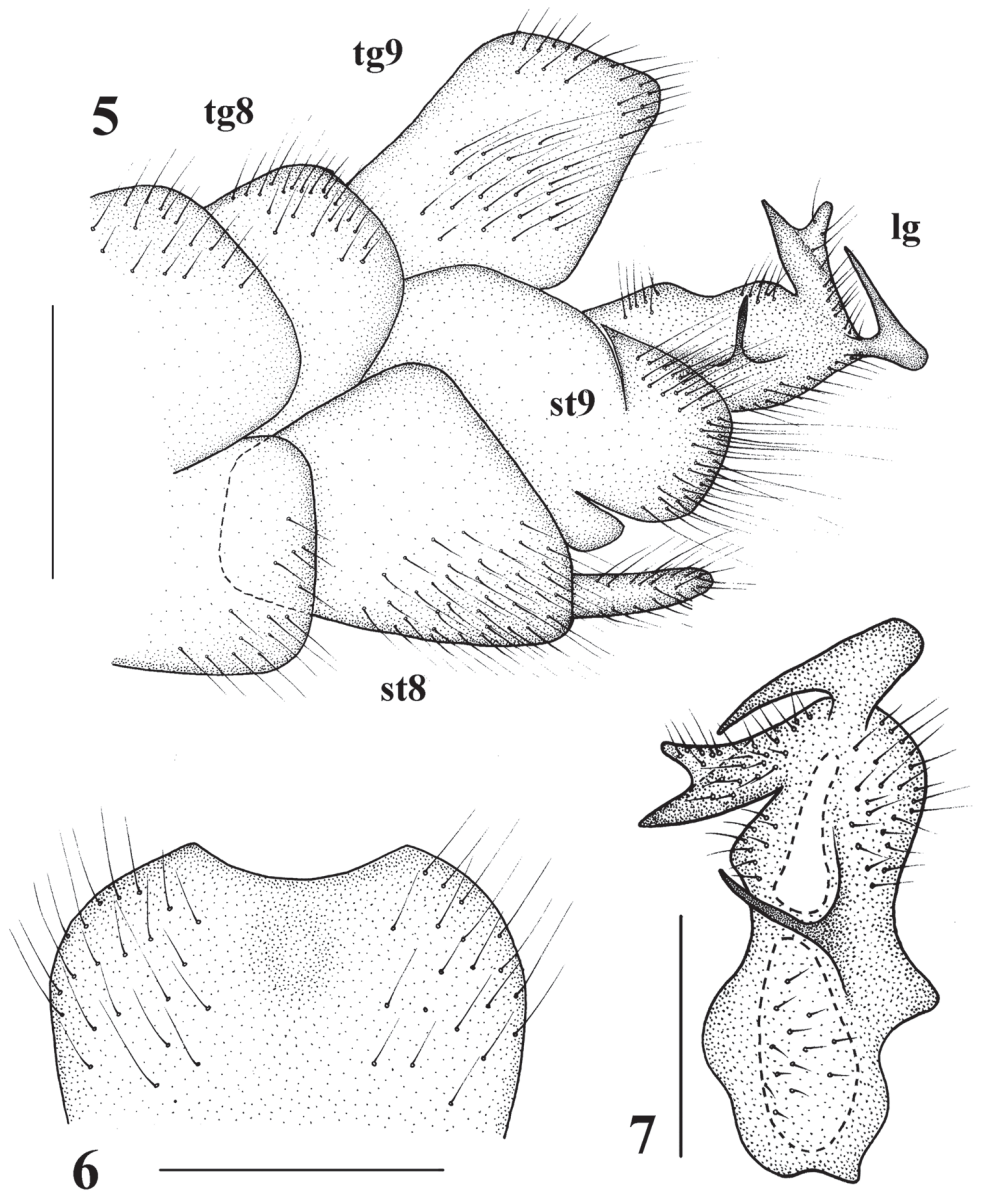
Tipula (Formotipula) melanomera gracilispina Savchenko, male **5** hypopygium, lateral view **6** ninth tergite, dorsal view **7** clasper of gonostylus, lateral view. tg 8 = eighth tergite, tg 9 = ninth tergite, st 8 = eighth sternite, st 9 = ninth sternite, lg = lobe of gonostylus. Scale bars: 1.0 mm (**5, 6**); 0.5 mm (**7**).

Hypopygium (Figs 5–7). Posterior margin of ninth tergite with a shallow, U-shaped notch. Posterior margin of eighth sternite with a pair of digitiform appendages. Clasper of gonostylus complex (Fig. 7), beak with triangular, membranous dorsal lobe, dorsal crest with a sickle-shaped dorsal process, basal beak and posterior crest with short setae; face of dististyle with a slender, acute, upwardly tilted spine.

Female (*n* = 3): Body length 13–15 mm, wing length 14–16 mm, antenna length 3–4 mm.

Female resembles male in head and thorax, except abdomen plump. Eighth tergite and eighth sternite black throughout with black setae. Ninth tergite, ninth sternite, and tenth tergite dark black, with black setae (Fig. 2).

**Figures 8–10.**
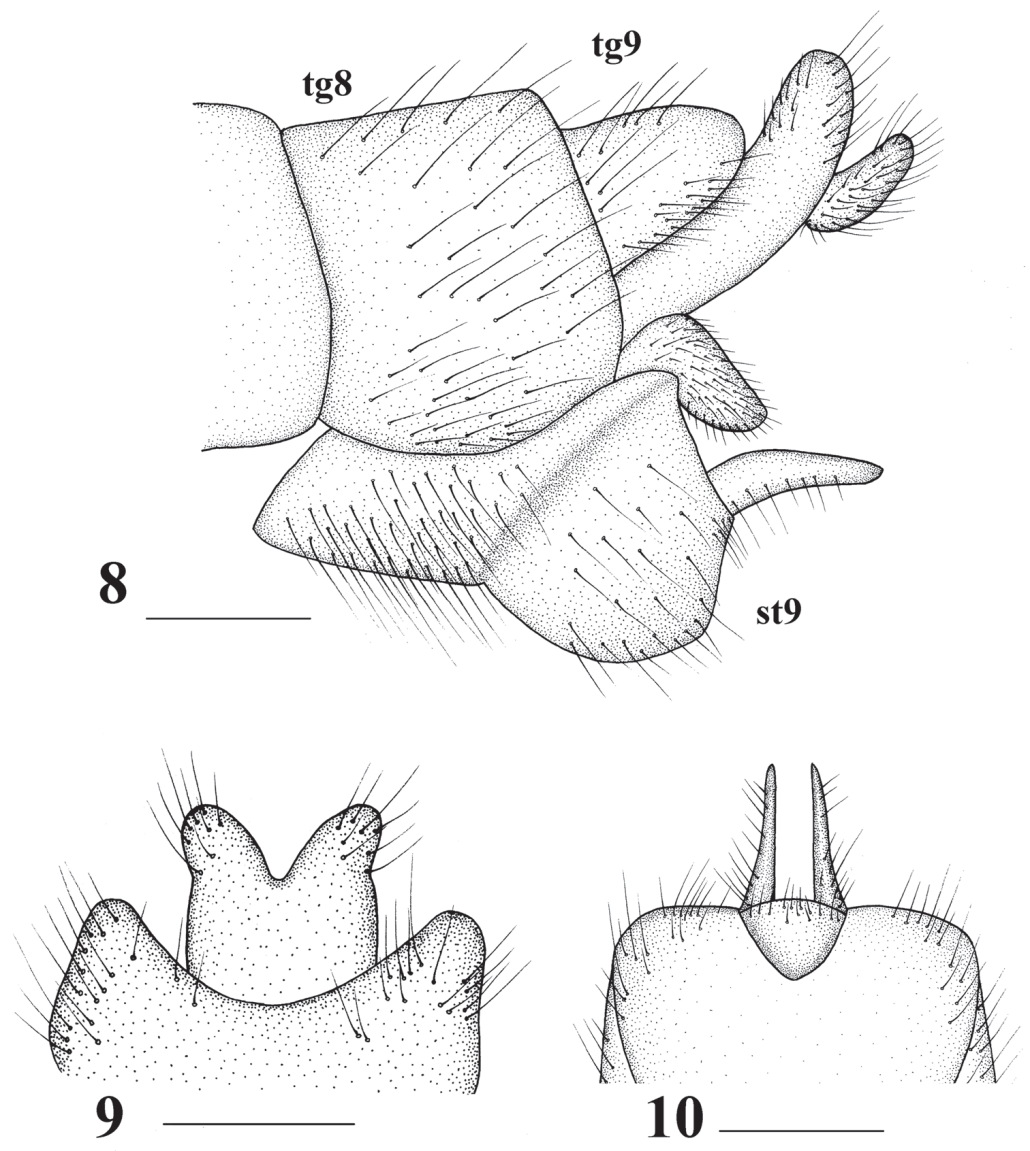
Tipula (Formotipula) melanomera gracilispina Savchenko, female **8** ovipositor, lateral view **9** ninth tergite and cerci, dorsal view **10** hypovalves, ventral view. tg 8 = eighth tergite, tg 9 = ninth tergite, st 9 = ninth sternite. Scale bars: 0.5 mm (**8–10**).

Ovipositor (Figs 8–10). Ninth tergite with two lobes separated by V-shaped, median emargination; each lobe with long black setae at tip. Ninth sternite slender. Cerci short, fleshy, apically obtuse. Tenth sternite flat with setae. Hypovalves small, sclerotized, sharply pointed, with black setae.

##### Material examined.

3 males 1 female (CAU), Laos: Houaphanh, Sam Nuea, Hvay Ma, 2017.VI.8, Liang Wang. 2 males 2 females (CAU), Laos: Attapeu, Sok. Samakhi Vay N. B., 2017.VI.18, Liang Wang.

##### Distribution.

China (Guizhou, Yunnan), Laos (Houaphanh).

#### 2. Tipula (Formotipula) melanomeramelanomera

Taxon classificationAnimaliaDipteraTipulidae

Walker, 1848

F05B3E8C-6853-5C28-BE94-8561B3036C16

Figs 11–14

Tipula melanomera Walker 1848: 68. Type locality: Nepal.
Tipula (Formotipula) melanomera : Edwards 1932: 238.

##### Diagnosis.

Hypopygium covered with dense long setae. Clasper of the gonostylus slender; beak short with a long tuber which directed upward; dorsal margin bearing a sickle-shaped prominence which directed anteriorly (Savchenko 1960: 888, fig. 1).

##### Material examined.

Recorded from Nepal and Upper Burma (BMNH).

##### Distribution.

India (Assam), Laos, Myanmar, Nepal.

**Figures 11–14.**
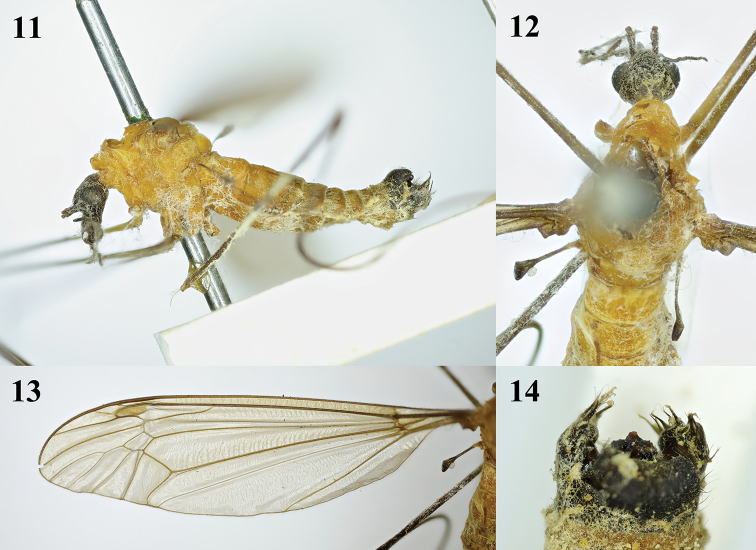
Tipula (Formotipula) melanomera melanomera Walker, male **11** habitus, lateral view **12** head and thorax, dorsal view **13** left wing **14** hypopygium, dorsal view.

#### 3. Tipula (Formotipula) melanopyga

Taxon classificationAnimaliaDipteraTipulidae

Edwards, 1926

BC99E99D-42D5-5B1A-A657-915435D80001

Figs 15–18

Tipula melanopyga Edwards 1926: 53. Type locality: Laos: Nam Mat.
Tipula (Formotipula) melanopyga : Li et al. 2013: 204.

##### Diagnosis.

Abdomen yellowish-white pruinose has blackish brown stripes, last two segments dark brown. Male ninth tergite has a slender hammer-shaped projection at middle. Clasper of the gonostylus with two short acute blackened spines (Li et al. 2013: 205, figs 16–18).

##### Type material examined.

Paratype, male, Laos: Nam Mat, 15 April 1918 (BMNH).

##### Distribution.

China (Yunnan), Laos.

**Figures 15–18.**
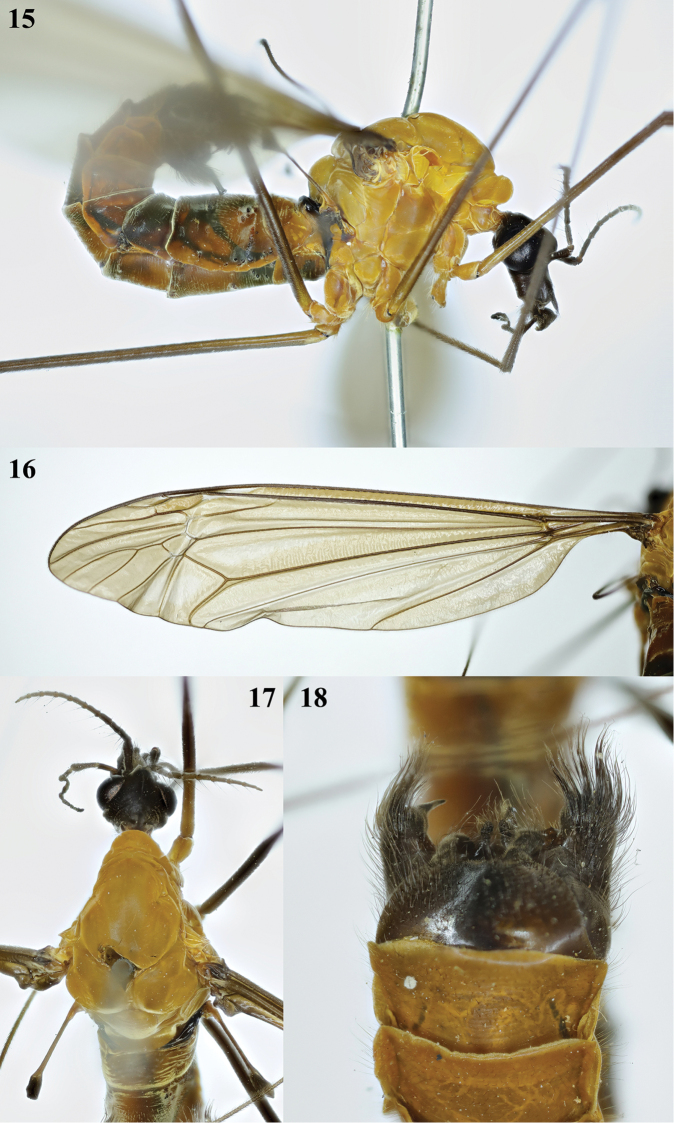
Tipula (Formotipula) melanopyga Edwards, male **15** habitus, lateral view **16** left wing **17** head and thorax, dorsal view **18** hypopygium, ventral view.

#### 4. Tipula (Nippotipula) champasakensis sp. nov.

Taxon classificationAnimaliaDipteraTipulidae

C9ED37BD-7F11-56E5-B8AC-BA8BAD091D99

http://zoobank.org/33A1651F-7FAD-4985-AC37-2E068BDAAC7C

Figs 19–22 , 23–27 , 28–33

##### Diagnosis.

Abdomen is reddish brown with thick brownish yellow setae. Eighth sternite extended backward, posterior margin has a deep median notch. Posterior margin of ninth tergite extended with sclerotized depression. Lobe of gonostylus is fleshy with a V-shaped depression at posterior margin. Clasper of gonostylus is duck-shaped.

##### Description.

Male (*n* = 8): body length 20–23 mm, wing length 16–18 mm, antenna length 5–6 mm.

**Figures 19–22.**
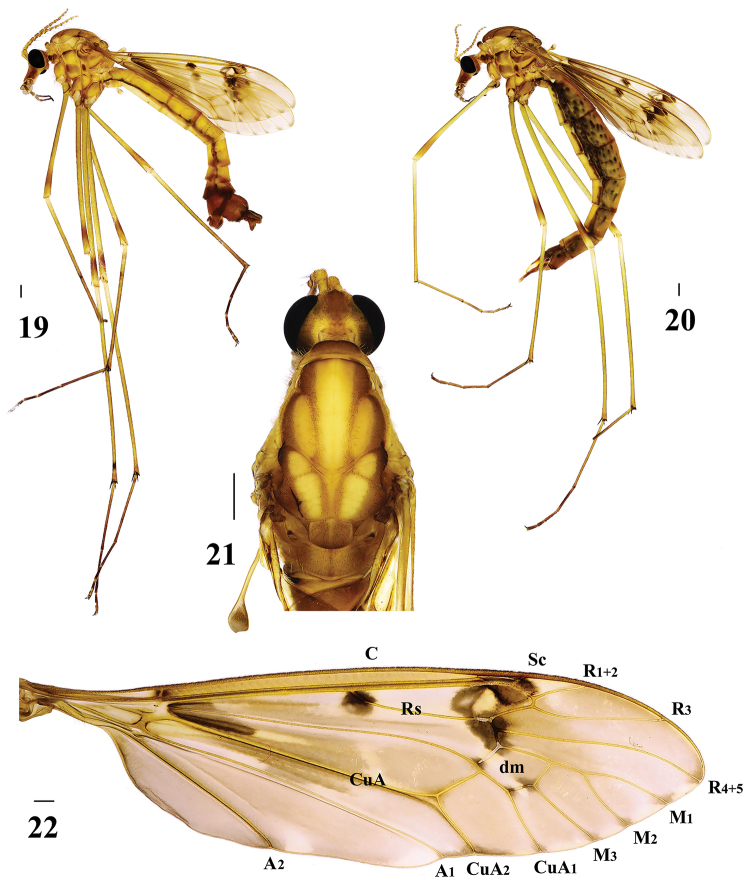
Tipula (Nippotipula) champasakensis sp. nov. **19** male habitus, lateral view **20** female habitus, lateral view; **21** male head and thorax, dorsal view **22** male right wing. Scale bar: 1.0 mm.

Head (Figs 19, 21). Mostly brownish yellow. Dorsal part of rostrum brownish yellow; nasus blacked; eyes dark black. Setae on head black. Antenna dark brown, except scape brownish and pedicel yellow; first flagellomere longest, slightly longer than scape. Proboscis mostly brownish, with black setae. Palpus greyish brown, with black setae.

Thorax (Figs 19, 21). Mainly brownish. Prescutum brownish yellow with three pale yellow stripes; median stripe broad basally with a light brown median line, lateral stripes oval, shorter than median stripe. Scutum brownish yellow, each lobe with two pale yellow stripes. Scutellum gray-yellow. Mediotergite yellow with long brownish yellow setae. Thoracic pleuron mostly brownish throughout, except middle of pleuron bright yellow or tinged brown. Setae on thorax brownish yellow. Legs coxae and trochanters grayish yellow, femora light brown, with subterminal dark band, tibiae, and tarsi brownish. Setae on legs black except those on coxae grayish yellow. Wings brownish yellow, with dark brown spots at the origin of Rs, M, and R_4+5_. Pterostigma dark brown with some macrotrichiae, Rs relatively long; cell m_1_ petiolate (Fig. 22). Halter length approximately 2.5 mm; halter stem brownish yellow, with brownish yellow setae; halter knob pale yellow (Fig. 21).

Abdomen (Fig. 19). Mainly brownish yellow. Abdominal segments 1–6 brownish yellow with yellow setae; segments 7 and 8 with brown setae. Hypopygium reddish brown, with brown setae.

**Figures 23–27.**
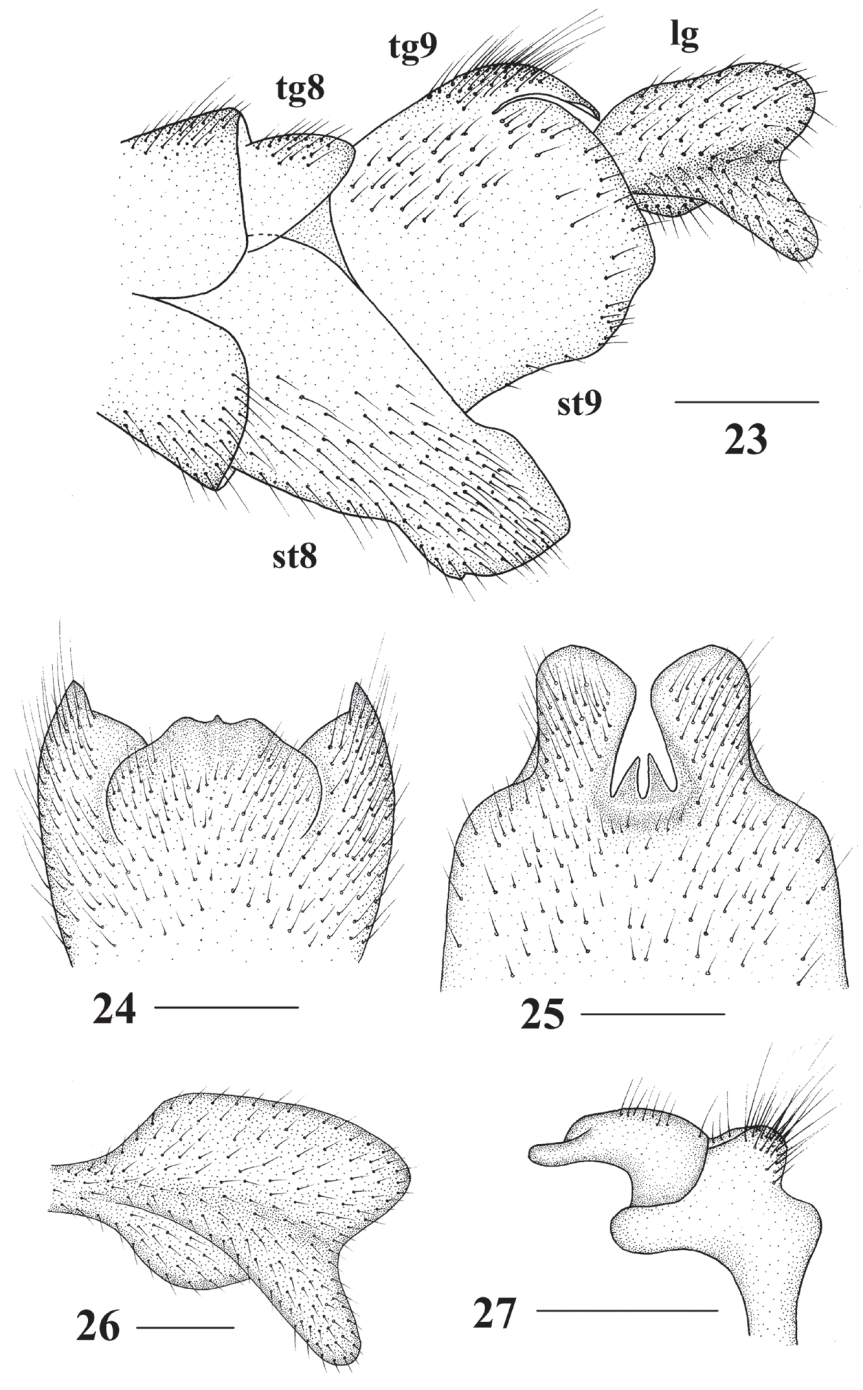
Tipula (Nippotipula) champasakensis sp. nov., male **23** hypopygium, lateral view **24** ninth tergite, dorsal view **25** eighth sternite, ventral view; **26** lobe of gonostylus, lateral view **27** clasper of gonostylus, lateral view. tg 8 = eighth tergite, tg 9 = ninth tergite, st 8 = eighth sternite, st 9 = ninth sternite, lg = lobe of gonostylus. Scale bars: 1.0 mm (**23–25**); 0.5 mm (**26, 27**).

Hypopygium (Figs 19, 23–27). Eighth sternite extended backward, posterior margin with a deep median notch and setae; two small, triangular processes present at bottom of notch. Posterior margin of ninth tergite extended with ossified depression; middle of extension with a small spiny protrusion. Ninth sternite with dark-brown setae laterally. Lobe of gonostylus fleshy with dense, dark-brown setae on outer side and dense, black, obtuse spinules on inner side, posterior margin with a V-shaped depression. Clasper of gonostylus duck-shaped; posterior crest with longer setae.

**Figures 28–33.**
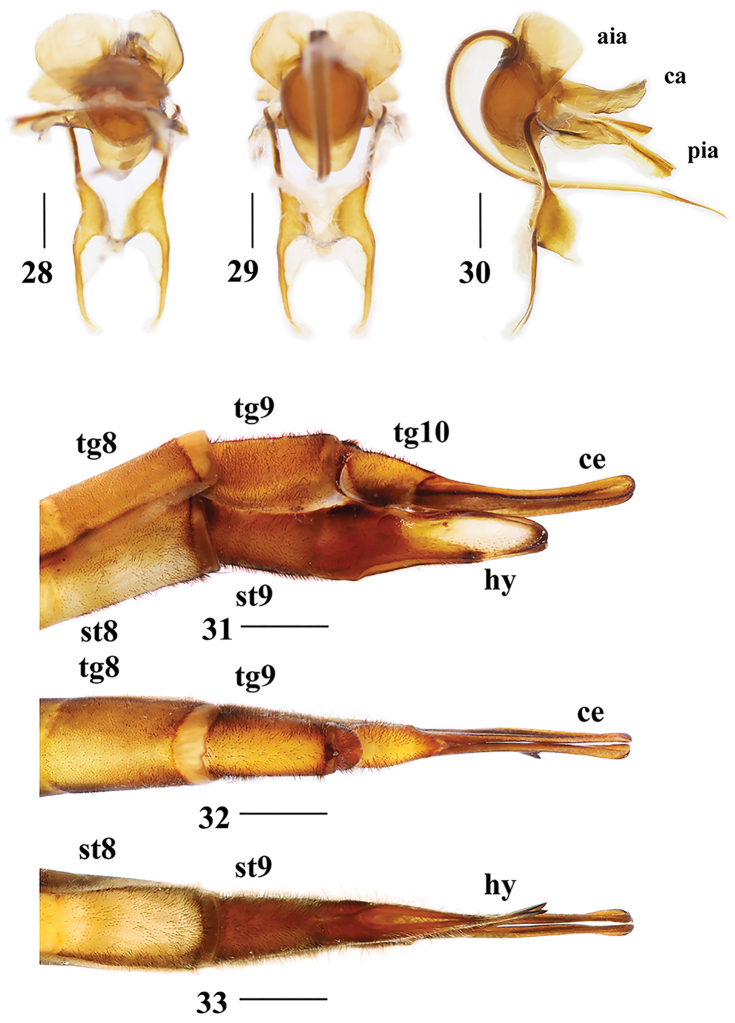
Tipula (Nippotipula) champasakensis sp. nov. **28**﻿﻿﻿–**30** male **28** semen pump, ventral view **29** semen pump, dorsal view **30** semen pump, lateral view **31–33** female **31** ovipositor, lateral view **32** ovipositor, dorsal view **33** ovipositor, ventral view. tg 8 = eighth tergite, tg 9 = ninth tergite, tg 10 = tenth tergite, st 8 = eighth sternite, st 9 = ninth sternite, aia = anterior immovable apodeme, pia = posterior immovable apodeme, ca = compressor apodeme, ce = cercus, hy = hypovalva. Scale bars: 0.5 mm (**28–30**); 1.0 mm (**31–33**).

Semen pump (Figs 28–30). Posterior immovable apodeme (pia) reddish brown, rod-like, and directed backward, symmetrical on both sides. Compressor apodeme (ca) fan-shaped and directed ventrally. Anterior immovable apodeme (aia) wing-shaped. Other appendages complex, as shown in Figures 28–30.

Female (*n* = 2): body length 22–23 mm, wing length 17–18 mm, antenna length 5–6 mm.

Female resembles male in head and thorax, except abdomen plump (Fig. 20). Eighth tergite and eighth sternite reddish brown throughout. Ninth tergite, ninth sternite, and basal region of tenth tergite dark reddish brown (Figs 31–33).

Ovipositor reddish brown (Figs 31–33). Cercus narrowed toward tip. Hypovalve curved, broad apically.

##### Type material.

***Holotype***: male (CAU), Laos: Champasak, Soukhouma, Dond Hua Sao N. B., 2017.VI.19, Liang Wang (light trap). ***Paratypes***: 2 males 2 females (CAU), Laos: Champasak, Sabaidee Valley, 2017.VI.16, Liang Wang (light trap); 5 males (CAU), Laos: Champasak, Soukhouma, Dond Hua Sao N. B., 2017.VI.19, Liang Wang (light trap).

##### Distribution.

Laos (Champasak).

##### Etymology.

The species is named after Champasak Province, where the type locality is located.

##### Remarks.

This new species is somewhat similar to T. (N.) coquilletti Enderlein, 1912 from Japan and T. (N.) sinica Alexander, 1935 from China (Zhejiang) in having a similarly shaped hypopygium, but it can be separated from these species by the shape of the eighth sternite and ninth tergite, posterior margin of eighth sternite with V-shaped notch which with two long triangular processes at bottom, and posterior margin of ninth tergite with a small spiny protrusion at middle. In T. (N.) coquilletti, the posterior margin of eighth sternite is without processes and V -shaped depression, and the posterior margin of the ninth tergite has a V-shaped notch. In T. (N.) sinica, the eighth sternite is without processes and V-shaped depression, and the posterior margin of the ninth tergite is extended with a sclerotized U-shaped depression.

#### 5. Tipula (Platytipula) sessilis

Taxon classificationAnimaliaDipteraTipulidae

Edwards, 1921

0E4A13C9-28FA-50C2-9350-7E23A44BE72F

Figs 34–37

Tipula sessilis Edwards 1921: 110 (as new name for Pachyrhina demarcata Brunetti, 1912).
Pachyrhina demarcata Brunetti 1912: 344. Type locality: India: Darjiling.
Tipula xanthopleura Edwards 1928: 698. Type locality: India: Kumaon, Muktesar.
Tipula (Schummelia) pergrata Alexander 1936b: 171. Type locality: India: Assam, Khasi Hills, Cherrapunji.
Tipula (Platytipula) xanthopleura : Savchenko 1961: 67.
Tipula (Schummelia) sessilis : Alexander and Alexander 1973: 57.
Tipula (Schummelia) xanthopleura : Alexander and Alexander 1973: 57.
Tipula (Schummelia) demarcata : Joseph 1974: 251.

##### Diagnosis.

Thoracic prescutum has three brown stripes; wing is brownish yellow and with brown pterostigma; Rs is a little longer than R_2+3_ and relatively straight; petiole of cell m_1_ is short. Abdomen is brownish yellow; notch of ninth sternite has a depressed semicircular lobe; lobe of gonostylus slender and gradually narrowed to obtuse tip, before the apex slightly narrower; clasper of gonostylus is a shallow, beak blackened, surface with abundant minute setae, lower lobe well developed (Alexander 1936b: 171, pl. 2, fig. 25).

##### Type material examined.

Holotype, male, India: Kumaon, Muktesar, Khasi Hills, Cherrapunji, 1 April 1922 (T. B. Fletcher) (BMNH).

##### Distribution.

China (Xizang), India (Assam, Uttarakhand, Uttar Pradesh, W Bengal), “Indochina” (= ?Laos), Tajikistan.

**Figures 34–37.**
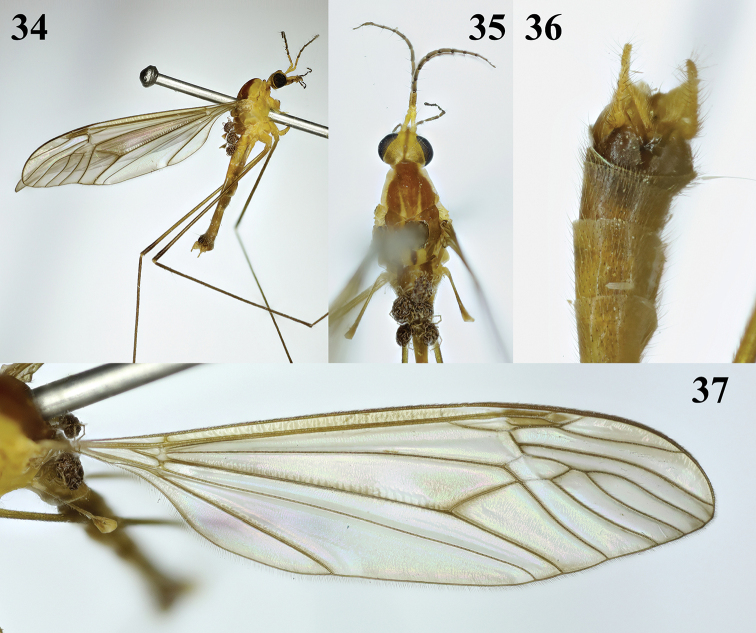
Tipula (Platytipula) sessilis Edwards, male **34** habitus, lateral view **35** head and thorax, dorsal view **36** hypopygium, dorsal view **37** right wing.

#### 6. Tipula

Taxon classificationAnimaliaDipteraTipulidae

(unplaced) reposita Walker, 1848

F46DF04F-CCFC-50B3-AB3F-90B1900076CC

Figs 38–43 , 44–48 , 49–52

Tipula reposita Walker 1848: 67. Type locality: Nepal.
Tipula brevis Brunetti 1918: 270. Type locality: India (Assam: Shillong)
Tipula brevis Edwards 1924: 307.
Tipula reposita Edwards 1926: 53. Locality: Laos: (Tong La).
Tipula (Vestiplex) brevis Alexander 1963: 23.
Tipula (Vestiplex) brevis Joseph 1974: 269.
Tipula (Vestiplex) reposita Starkevich et al. 2015: 122. Locality: India, Laos, Nepal, and Thailand (Chiang Mai).
Tipula reposita Walker, 1848. Pilipenko et al. 2019. Locality: Thailand (Chiang Mai).
Tipula (Oreomyza) cladomera Alexander 1936a: 230. Type locality: China: “Szechwan: Wan-hsien” (= Chongqing: Wanzhou). syn. nov.

##### Diagnosis.

Rs is relatively long and cell m_1_ is petiolate. Tip of eighth sternite has long dense thick setae. Ninth tergite has a U-shaped depression. Lobe of gonostylus is subtriangular and posterior margin has a shallow V-shaped incision with a black sclerotized protuberance. Clasper of gonostylus is small and beak-like.

##### Description.

Male (*n* = 3): Body length 12–13 mm, wing length 13–14 mm, antenna length 4–4.5 mm.

**Figures 38–43.**
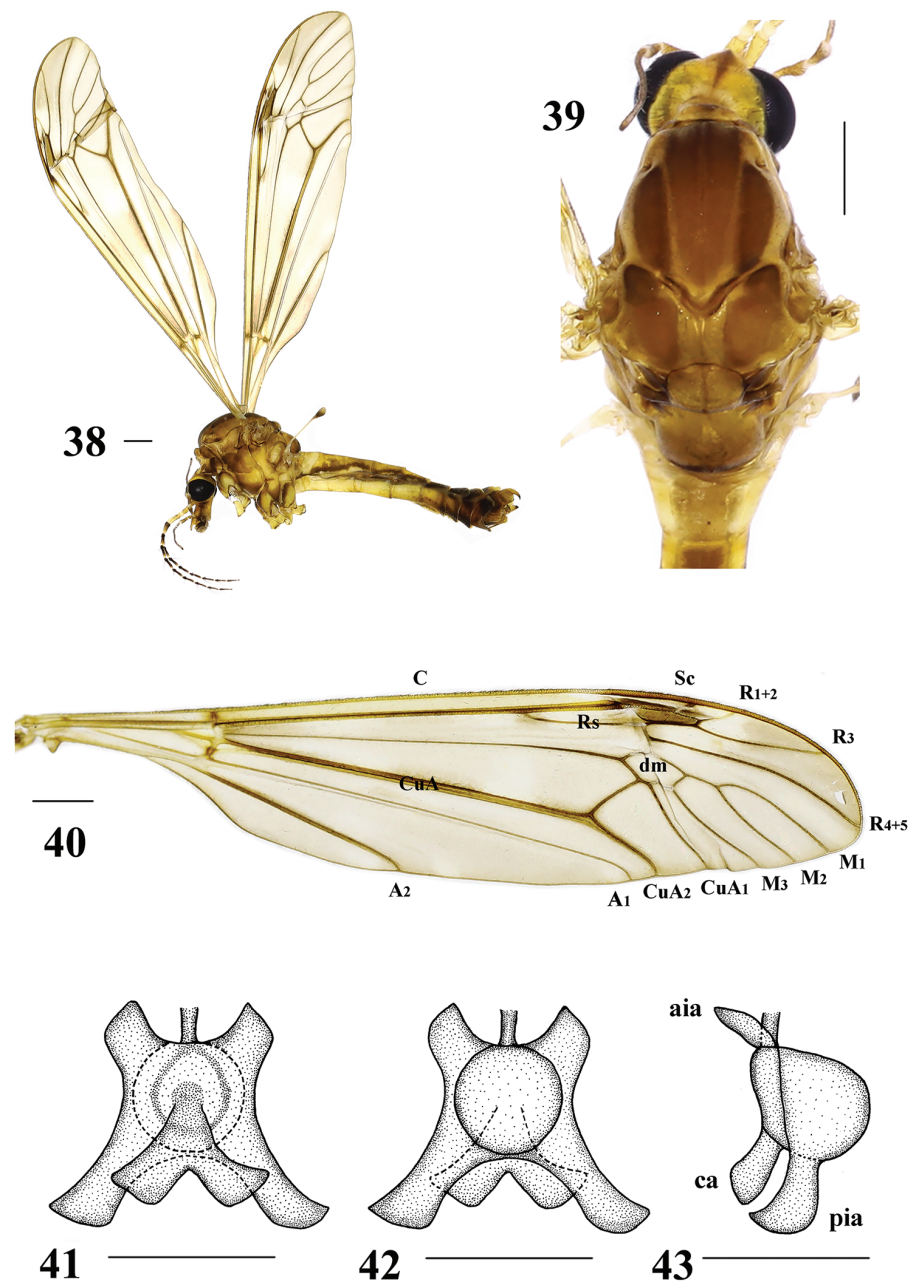
*Tipula* (unplaced) *reposita* Walker, male **38** habitus, later view **39** head and thorax, dorsal view **40** right wing **41** semen pump, ventral view **42** semen pump, dorsal view **43** semen pump, lateral view. aia = anterior immovable apodeme, pia = posterior immovable apodeme, ca = compressor apodeme. Scale bars: 1.0 mm (**38–40**); 0.5 mm (**41–43**).

Head (Figs 38, 39). Mostly brownish yellow. Dorsal part of rostrum brownish yellow, with distinct long nasus. Eyes black. Setae on head black. Antenna dark brown except scape brownish and pedicel yellow; first flagellomere longest, slightly longer than scape. Proboscis mostly brown with black setae; palpus greyish brown, with black setae.

Thorax (Figs 38, 39). Mainly brownish. Pronotum yellow with a light brown spot at middle. Prescutum yellow with three brown stripes; median stripe narrowed basally with a light brown median line; lateral stripes oval, a little shorter than median stripe. Scutum yellow, each lobe with a brown stripe. Scutellum yellow with a brown margin. Mediotergite yellow with a brown area near hind margin. Pleuron brownish yellow. Setae on thorax brownish yellow. Legs with coxae and trochanters yellow; femora light brown with dark tips; tibiae and tarsi dark brown. Setae on legs black, except those on coxae yellow. Wings light brown; pterostigma dark brown with some macrotrichia; posterior margin of cell cua_1_ as wide as base; Rs relatively long, cell m_1_ petiolate (Fig. 40). Halter length approximately 2.5 mm; halter stem pale yellow; halter knob brownish gray, with brownish setae.

Abdomen (Fig. 38). Mainly brownish yellow. Abdominal segments 1–5 brownish yellow with yellow setae; remaining segments dark brown with light brown setae.

**Figures 44–48.**
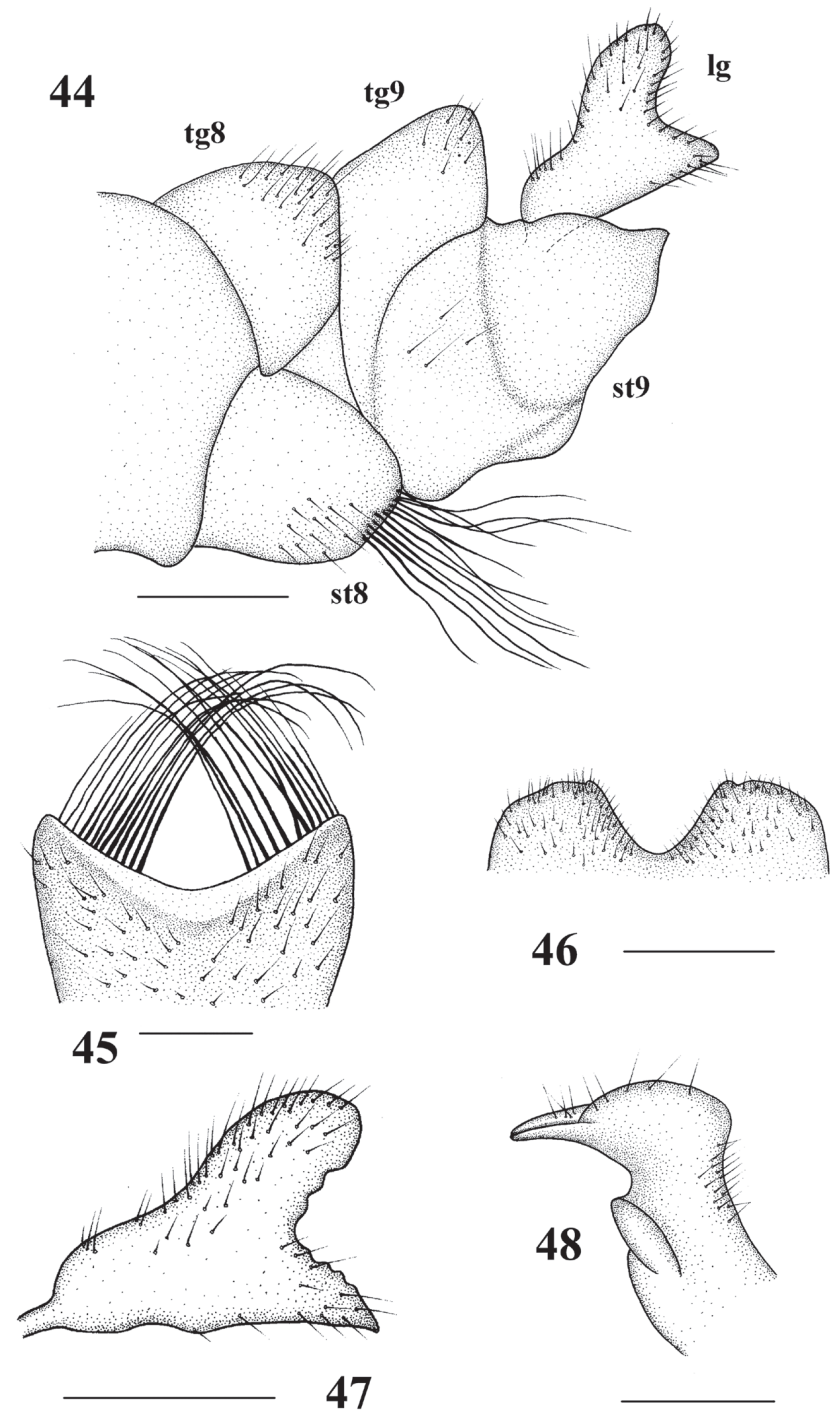
*Tipula* (unplaced) *reposita* Walker. Male **44** hypopygium, lateral view **45** eighth sternite, ventral view **46** ninth tergite, dorsal view **47** lobe of gonostylus, lateral view **48** clasper of gonostylus, lateral view. tg 8 = eighth tergite, tg 9 = ninth tergite, st 8 = eighth sternite, st 9 = ninth sternite, lg = lobe of gonostylus. Scale bars: 0.5 mm (**44–47**); 0.25 mm (**48**).

Hypopygium (Figs 44–48). Eighth sternite with dense long thick setae at tip. Ninth sternite sclerotized, convex. Ninth tergite with a U-shaped depression, laterally with yellow setae. Hypopygium mostly brownish yellow, lobe of gonostylus triangular, slightly curved, broadened apically, posterior margin with shallow V-shaped incision and with black sclerotized protuberance. Clasper of gonostylus small, beak-like.

Semen pump (Figs 41–43). Posterior immovable apodeme (pia) brownish yellow, rod-like, and directed backward. Compressor apodeme (ca) ginkgo-leaf-shaped and directed ventrally. Anterior immovable apodeme (aia) triangular.

##### Material examined.

3 males (CAU), Laos: Houaphanh, Sam Nuea, B. Meuang Lied, 2017.VI.9, Liang Wang (light trap); *T.* (unplaced) *cladomera*: holotype, male, China: “Szechwan: Wan-hsien” (= Chongqing: Wanzhou), September 14, 1921, American Museum of Nature History, accession no. 23974 (USNM).

##### Distribution.

China (Chongqing), India (Assam), Laos (Tong La, Houaphanh), Nepal, Thailand (Chiang Mai).

##### Remarks.

*Tipula* (unplaced) *cladomera* was proposed by Alexander (1936a, as T. (Oreomyza) cladomera)) and some morphological differences were indicated. According to Alexander (1936a), males of *T.* (unplaced) *cladomera* are characterized by the following features: hypopygium with the lobe of gonostylus very large and of unusual shape, expanded outwardly, the apex with a U-shaped notch which forms two conspicuous lobes; eighth sternite with nine or ten very coarse setae on either side of midline of the caudal margin (Fig. 51; Alexander 1936a: 230; pl. 2, figs 25, 26). After comparison of the type specimens of *T.* (unplaced) *cladomera* Alexander, 1936 with T. (Vestiplex) reposita Walker, 1848 and our specimens from Laos, we found that those specimens do not have clear differences and are characterized by the same features, even though the males of *T.* (unplaced) *cladomera* have no shorter cross-vein between cell dm and cell cua_1_ (Fig. 50; Alexander 1936a: pl. 1, fig. 5), and T. (Vestiplex) reposita does have shorter cross-vein between cell dm and cell cua_1_ (Brunetti in Joseph 1974: 268, fig. 109). Altogether, through examining many specimens, including holotypes and paratypes, and the literature, we attribute such differences as intraspecific variation and consider these two species to be the same and T. (O.) cladomera to be a junior synonym of *T.* (unplaced) *reposita*. This species used to be placed in the Tipula subgenus Vestiplex, although in our opinion, such an arrangement is incorrect because of the unique male genital complex. *Tipula* (unplaced) *reposita* Walker, 1848 does not belong to any existing subgenus and is to be considered unplaced.

**Figures 49–52.**
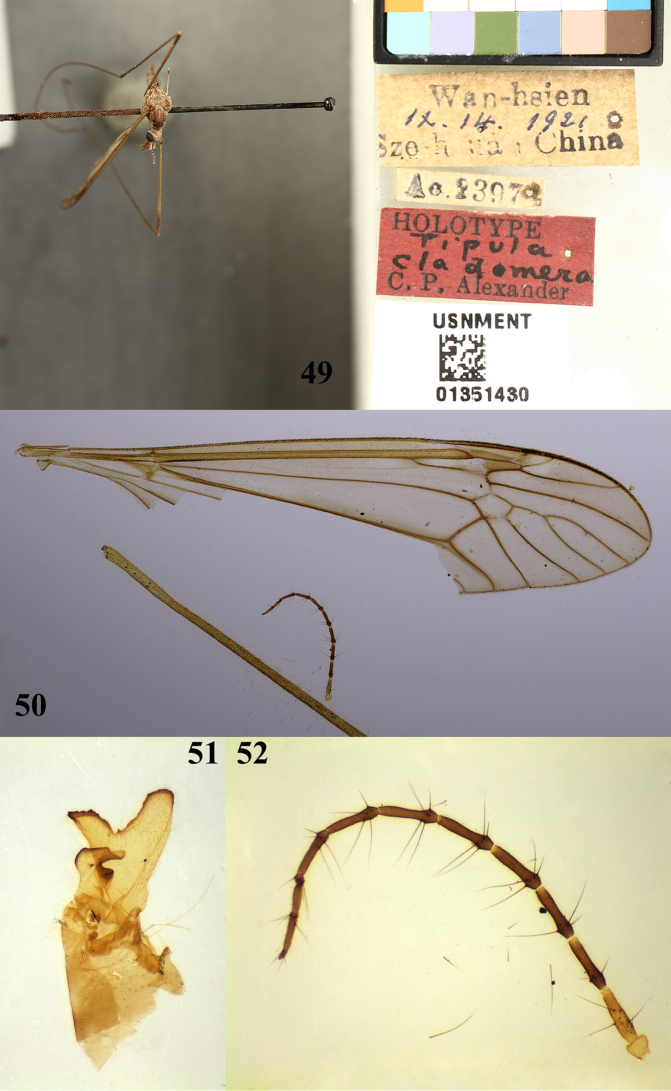
*Tipula* (unplaced) *reposita* Walker., holotype male **49** habitus, dorsal view **50** right wing **51** lobe of gonostylus and clasper of gonostylus, lateral view **52** antenna.

## Supplementary Material

XML Treatment for Tipula (Formotipula) melanomeragracilispina

XML Treatment for Tipula (Formotipula) melanomeramelanomera

XML Treatment for Tipula (Formotipula) melanopyga

XML Treatment for Tipula (Nippotipula) champasakensis

XML Treatment for Tipula (Platytipula) sessilis

XML Treatment for Tipula
